# Disorders of sex development: a genetic study of patients in a multidisciplinary clinic

**DOI:** 10.1530/EC-14-0085

**Published:** 2014-10-13

**Authors:** Luigi Laino, Silvia Majore, Nicoletta Preziosi, Barbara Grammatico, Carmelilia De Bernardo, Salvatore Scommegna, Anna Maria Rapone, Giacinto Marrocco, Irene Bottillo, Paola Grammatico

**Affiliations:** Department of Molecular Medicine, Medical Genetics, San Camillo-Forlanini Hospital, Sapienza University, A.O. San Camillo-Forlanini, Padiglione Morgagni, I piano, UOC Laboratorio di Genetica Medica, Circonvallazione Gianicolense 87, Rome, 00152, Italy; 2 Department of Pediatrics and Hematology, San Camillo-Forlanini Hospital, A.O. San Camillo-Forlanini, Padiglione Baccelli, II piano, Pediatria ed Ematologia Pediatrica, Circonvallazione Gianicolense 87, Rome, 00152, Italy; 3 Psychology Department, San Camillo-Forlanini Hospital, A.O. San Camillo-Forlanini, Dipartimento di Pscicologia, Circonvallazione Gianicolense 87, Rome, Italy; 4 Department of Pediatric Surgery, San Camillo-Forlanini Hospital, A.O. San Camillo-Forlanini, Padiglione Baccelli, II piano, Pediatria ed Ematologia Pediatrica, Circonvallazione Gianicolense 87, Rome, 00152, Italy

**Keywords:** disorders of sex development, DSD, disorders of ovarian development, disorders of testicular development

## Abstract

Sex development is a process under genetic control directing both the bi-potential gonads to become either a testis or an ovary, and the consequent differentiation of internal ducts and external genitalia. This complex series of events can be altered by a large number of genetic and non-genetic factors. Disorders of sex development (DSD) are all the medical conditions characterized by an atypical chromosomal, gonadal, or phenotypical sex. Incomplete knowledge of the genetic mechanisms involved in sex development results in a low probability of determining the molecular definition of the genetic defect in many of the patients. In this study, we describe the clinical, cytogenetic, and molecular study of 88 cases with DSD, including 29 patients with 46,XY and disorders in androgen synthesis or action, 18 with 46,XX and disorders in androgen excess, 17 with 46,XY and disorders of gonadal (testicular) development, 11 classified as 46,XX other, eight with 46,XX and disorders of gonadal (ovarian) development, and five with sex chromosome anomalies. In total, we found a genetic variant in 56 out of 88 of them, leading to the clinical classification of every patient, and we outline the different steps required for a coherent genetic testing approach. In conclusion, our results highlight the fact that each category of DSD is related to a large number of different DNA alterations, thus requiring multiple genetic studies to achieve a precise etiological diagnosis for each patient.

## Introduction

Sex development is a multistep process under genetic control, implying a delicate network of molecular events that direct both the bi-potential gonads to become either a testis or an ovary (sex determination), and the consequent divergent differentiation of internal ducts and external genitalia (sex differentiation). Correct dimorphic sex determination and differentiation achievement can be disrupted by a large number of genetic and non-genetic factors altering any of the molecular signals that specify sex-specific development of sex organs or endocrine function. The term disorders of sex development (DSD) embraces all the medical conditions characterized by an atypical chromosomal, gonadal, or phenotypical sex (1). Thus, a wide number of pathologies are included under the same DSD definition; they show different frequencies and their severity ranges from genital anomalies that do not impair sexual definition or functionality, such as hypospadias, to conditions characterized by sexual ambiguity or discordance between chromosomal and internal or external sex anatomy. In 2006, Hughes *et al*. (1) proposed the latest recommended classification of DSD, based on the sex chromosomal findings and on the step of gonadal development or phenotypic differentiation in which the alteration had occurred. Current understanding of the genetic control of sex development is still incomplete, resulting a low probability of determining the molecular definition of the causal defect in many of the patients with DSD. Anyhow, proper and thorough clinical evaluation and laboratory investigations are the necessary procedures for obtaining the most accurate diagnostic definition. This can more efficiently be achieved through a multidisciplinary assessment of patients performed by different dedicated specialists with long-standing experience and both pediatric and adult practice. In this study, we describe the results of studies carried out on 88 patients with DSD evaluated and followed in the outpatient ‘Centre for diagnosis, care and treatment of DSD’ at San Camillo Forlanini Hospital, Sapienza University of Rome. In total, we found a genetic alteration in 56 out of 88 cases, leading to the correct clinical classification of every patient. Each category of DSD was found to be related to a large number of different DNA alterations, thus requiring multiple genetic studies to possibly achieve a precise etiological diagnosis in every patient.

## Subjects and methods

### Patients

A cohort of 88 individuals, aged from 1 day to 41 years affected by non-syndromic DSD, were fully evaluated at the DSD Centre of San Camillo-Forlanini Hospital, Rome (Italy), by an experienced multidisciplinary team including a pediatric surgeon, a pediatric endocrinologist, a clinical psychologist, and a clinical geneticist. Patients were identified on the basis of ambiguous genitalia or discordance among chromosomal, gonadal and/or phenotypic sex, or apparently minor genital abnormalities (Table 1, modified from Hughes *et al*. (1)). Patients in whom the presence of additional anomalies, such as dysmorphic features and skeletal or visceral abnormalities, was detected were excluded from the cohort with the exception of the three syndromic patients (cases 6, 27, and 32) included in the study. For each patient, hormonal, imaging, and genetic investigations were carried out. Individuals showing 45,X, 47,XXX, and 47,XXY karyotypes, as well as 46,XX patients with gonadal dysgenesis and 46,XY patients with minor genital anomalies that did not give rise to any doubts about sex assignment, were excluded from the present report. In contrast, 46,XX female patients with developmental anomalies of the Müllerian duct were included in this cohort. In all cases, a stepwise clinical diagnostic approach for evaluation was conducted and psychological support was constantly provided to both patients and their families. The genetic analyses included the study of *AR*, *AMH*, *CYP11B1*, *CYP21A2*, *DHH*, *DMRT1*, *NR0B1*, *NR5A1*, *RSPO1*, *SHOX*, *SOX9*, *SRD5A2*, *SRY*, and *WNT4* genes chosen on the basis of the data reported in the literature (2, 3, 4, 5). The genes analyzed for each patient were selected according to the DSD category. All enrolled individuals gave informed consent for DNA analyses, approved by local ethics committees in accordance with the guidelines of Italian Society of Human Genetics (SIGU).

### Karyotyping

Metaphase spreads were obtained from blood lymphocytes using standard procedures. Chromosome analysis was performed using standard G-bands by trypsin using Giemsa (GTG)-banding techniques on cultured lymphocytes.

### Testing the presence or absence of *SRY* gene

Recognition of the *SRY* sequence was carried out on genomic DNA through polymerase chain reaction (PCR) amplification with specific *SRY* and control (*ZP3*) gene primers as described by Cui *et al*. (6).

### Fluorescent *in situ *hybridization

*SRY* translocations in 46,XX male patients and *DMRT1* deletions were investigated by fluorescent *in situ *hybridization (FISH) using two specific probes selected from a public database (http://genome.ucsc.edu), respectively, for *SRY* and *DMRT1* genes.

### Direct sequencing

The search for DNA point mutations in *SRY, DHH, NR5A1, SRD5A2, AR, AMH, CYP21A2, CYP11B1, RSPO1*, and* WNT4 *genes was carried out by PCR followed by direct sequencing. *SRD5A2, CYP21A2*, *AMH*, and *CYP11B1 *were analyzed as described previously (7, 8, 9). Primer sequences and annealing temperatures employed for amplification of *SRY, DHH, NR5A1, AR, RSPO1, *and* WNT4 *coding sequences are listed in Supplementary Table 1, see section on supplementary data given at the end of this article. Primers were designed in order to also detect possible splicing defects. Sanger sequencing was carried out using a BigDye Terminator v1.1 Cycle Sequencing Kit (Thermo Fisher Scientific, Waltham, MA, USA) on a 3500xl Genetic Analyzer (Thermo Fisher Scientific). Forward and reverse sequences were analyzed and compared with each gene's mRNA reference sequence: *SRY* (NM_003140.1), *DHH* (NM_010144), *NR5A1* (NM_004959), *SRD5A2* (NM_000348.3), *AR* (NM_000044.2), *AMH* (NM_000479), *CYP11B1* (NM_000497), *CYP21A2* (NM_000500), *RSPO1* (NM_001038633.2), and *WNT4* (NM_030761). All novel missense and splicing mutations were searched for in 200 control chromosomes from unaffected subjects.

### *CYP21A2* large deletions/conversions analysis

Investigation for large deletions/conversions affecting the *CYP21A2* gene was conduced as described by Lee *et al*. (10).

### Multiplex ligation-dependent probe amplification (MLPA) analysis

Screening for single and multi-exonic deletions/duplications affecting *SOX9, NR0B1, NR5A1, SRY, CYP21A2*, and *WNT4* genes was carried out using the MLPA SALSA P185-B2 Intersex (version 05; April 22, 2011) (MRC Holland, Amsterdam, The Netherlands), following the manufacturer's instructions. Information on probe sequences can be freely obtained from the MRC Holland website (www.mlpa.com). Relative quantification of copy number mutations was carried out using the Coffalyser Software (MRC Holland). DNA samples showing such a reduction or increase in the MLPA peak area values were reanalyzed by the same MLPA procedure. Only the samples showing consistent results between the two experiments were considered positive for a copy number alteration.

### Real-time PCR

Copy number changes in *NR0B1* genes identified by MLPA analyses and single/multi-exonic deletions/duplications of the *SHOX *gene were investigated using SYBR Green-based experiments on an ABI7900 HT Fast Real Time PCR System (Thermo Fisher Scientific). Primers were designed using the Primer Express 3.0 Software (Thermo Fisher Scientific; Supplementary Table 2, see section on supplementary data given at the end of this article). The reference gene telomerase reverse transcriptase (*TERT*) was simultaneously quantified in a separate tube for each specimen. For each primer pair, the reaction efficiency parameter (R2) was assessed by a standard-curve analysis as reported in Supplementary Table 2. Results for each sample were expressed as N-fold changes in copies of each test exon, and normalized to *TERT* relative to the copy number of the test exon in the calibrator DNA, according to the following equation: amount of target=2^−^^ΔΔ^ ^Ct^ (11).

## Results

### Classification of patients with DSD

**Figure 1:**
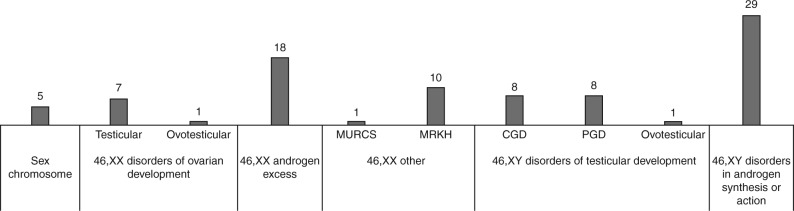
Etiological classification of 88 patients with DSD. After karyotyping and *SRY*-specific FISH analyses, the patients were classified using the classes suggested by Hughes *et al*. (1). MURCS, Müllerian aplasia, Renal aplasia, and Cervico-thoracic Somite dysplasia; MRKH, Mayer-Rokitansky-Kuster-Hauser; CGD, Complete Gonadal Dysgenesis; PGD, Partial Gonadal Dysgenesis.

Classical cytogenetic techniques were employed in order to categorize each patient into the correct DSD class according to the karyotype. Thirty-seven patients showed a 46,XX karyotype, while 46 patients had a 46,XY karyotype. Out of 88 patients, five (Table 2, cases 1 to 5) showed an aberrant karyotype with mosaicism involving numerical and/or structural abnormalities of sex chromosomes. Those patients were then classified as affected by sex chromosome DSD. *SRY* absence/presence test, successively performed on the seven males showing a 46,XX karyotype, identified the *SRY* gene in two patients (Table 2, cases 7 and 8). FISH analysis performed with a *SRY*-specific probe defined those patients’ karyotype as 46,XX.ish der(X)(X;Y)(p22.3;p11.3)(SRY+), indicative of whole *SRY* gene translocation to the X chromosome in both cases. In accordance with karyotyping and FISH analyses, our cohort was determined to be composed as shown in Fig. 1. On the basis of subsequent clinical investigations, out of 88 patients, 29 (33%) were classified as carriers of a 46,XY disorder in androgen synthesis or action, 18 (20%) as carriers of a 46,XX disorder with androgen excess, 17 (19%) as carriers of 46,XY disorders of gonadal (testicular) development, 11 (13%) as affected by 46,XX DSD other, 8 (9%) as carriers of 46,XX disorders of gonadal (ovarian) development, and 5 (6%) as carriers of sex chromosome DSD.

### DSD 46,XX disorders of ovarian development

Both the five 46,XX testicular (*SRY*-negative) patients and the single 46,XX ovotesticular patient with DSD (Fig. 1) were first analyzed by MLPA for the presence of copy number imbalances affecting *SOX9, NR0B1, NR5A1, SRY, CYP21A2*,and *WNT4* genes. In a single individual with 46,XX testicular DSD (Table 2, case 9), we identified a heterozygous duplication encompassing *SOX9* exon 1. The five 46,XX testicular (*SRY*-negative) patients and the single 46,XX ovotesticular patient with DSD (Fig. 1) were then investigated for the presence of *RSPO1* point mutations. We identified the homozygous c.286+1G>A (p.I32_l95del) splicing mutation in case 6 (Table 2). This patient was affected by 46,XX ovotesticular DSD associated with palmoplantar hyperkeratosis, congenital bilateral corneal opacity, and strabismus and this has already been reported (12). In total, we did not find any genetic alteration in four out of seven 46,XX testicular patients with DSD.

### DSD 46,XX androgen excess

Eighteen 46,XX female patients with suspected adrenogenital syndrome derived from 21-hydroxylase enzyme deficiency (Fig. 1) were analyzed for *CYP21A2 *gene mutations. One of these patients, in whom the hormonal profile was indicative of a rare form of the disease, was secondly analyzed for *CYP11B1 *point mutations. In total, we found *CYP21A2* DNA alterations in 14 out of 18 (78%) patients (Table 2, cases 10–23). Among the seven different identified *CYP21A2 *mutations, the c.293-13C/A>G change was present in heterozygosity in six out of 14 patients and in the homozygous condition in patient 10 (Table 2). *CYP21A2 *large deletion/conversion was detected in heterozygosity in six out of 14 cases and in homozygosity in three out of 13 patients (Table 2). Out of 14 individuals, 8 (57%) were compound heterozygous for *CYP21A2* mutations, while six out of 14 (43%) were homozygous (Table 2). Parental DNA for cases 16 and 22 (Table 2) was available for determination of the origin of the mutations. *CYP21A2* del/conv was paternally derived in patient 16, while the mother harbored the c.293-13C/A>G mutation. Regarding patient 22, the c.920_921insT (p.L307insT) and the c.293-13C/A>G alterations were inherited, respectively, from the mother and the father. The result of the *CYP11B1 *test indicated that the single analyzed patient (Table 2, case 24) was homozygous for the p.G379V mutation. All the identified pathogenetic alterations have been reported previously (13, 14, 15, 16, 17, 18), except for *CYP21A2* c.365T>C (p.L122P) that was found to be novel.

### DSD 46,XX, other (Mullerian Renal Cervicothoracic Somite association and Mayer Rokitansky Küster Hauser syndrome

The single Mullerian Renal Cervicothoracic Somite (MURCS) and the ten Mayer Rokitansky Küster Hauser (MRKH) individuals (Fig. 1), demonstrated to be negative in a previously performed array comparative genomic hybridization analyses (data not shown), were analyzed for the presence of *WNT4* point mutations and for *SHOX* copy number imbalances. None of the patients were found to carry a *WNT4* alteration, while a duplication of *SHOX* exon 6 in the heterozygous state was found in two out of ten MTKH patients (Table 2, cases 25–26).

### DSD 46,XY disorders of testicular development

Seventeen 46,XY patients (Fig. 1) were studied for variations in the *SRY*, *NR5A1,* and *DHH *genes. We identified the heterozygous c.301C>G (p.L101V) *SRY* missense mutation in patient 28 (Table 2) manifesting complete gonadal dysgenesis. Three patients with partial gonadal dysgenesis were heterozygous carriers of three different *NR5A1 *mutations: c.691_699 dupCTACAGCTG (p.L231_L233dup) (19) (Table 2, patient 29), c.86C>T (p.T29M) (Table 2, patient 30), and c.872_874delTGG (p.V291del) (Table 2, patient 31). None of the *DHH* point mutations were found in the analyzed patients. Following *SRY*, *NR5A1,* and *DHH *screening, the 13 out of 17 negative cases were tested for copy number alterations in *NR0B1*, *WNT4* and *DMRT1* genes. Patient 27 (Table 2) showing complete gonadal dysgenesis carried a heterozygous whole *NR0B1* gene duplication. We identified a heterozygous deletion encompassing the *DMRT1* gene in patient 32 (Table 2) manifesting partial gonadal dysgenesis. Finally, we did not detect a *WNT4* imbalance in any of the investigated patients.

### DSD 46,XY disorders in androgen synthesis or action

**Figure 2:**
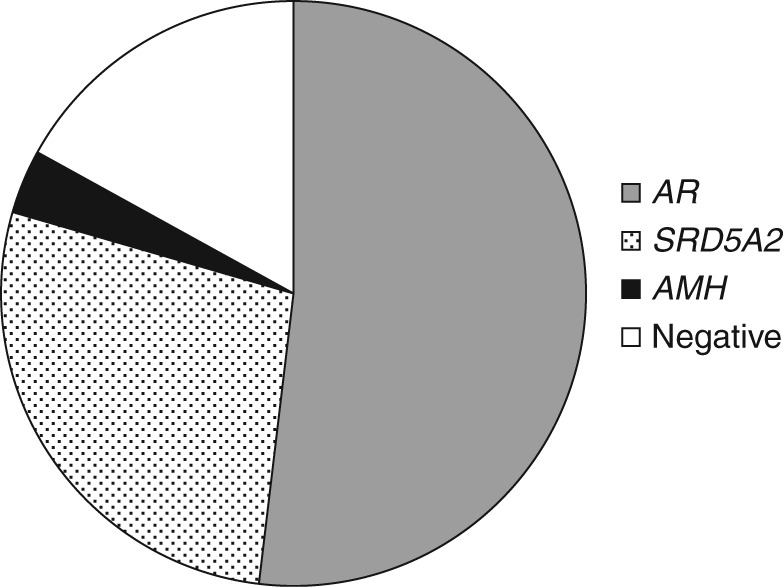
Point mutation analysis of *AR*, *SRD5A2,* and *AMH* genes in 29 patients with 46,XY DSD disorders in androgen synthesis or action. Out of 29 patients, 15 (52%) harbored a mutation in the *AR* gene, 8 (27%) in the *SRD5A2* gene, and 1 (3%) in the *AMH* gene. Out of the 29 patients, 5 (17%) did not show any point mutations in the coding sequences of the analyzed genes.

Sequence analysis of *AR* and *SRD5A2* genes was performed in 29 patients with suspected DSD 46,XY defects in the synthesis or action of androgens (Fig. 1). The *AMH* gene was sequenced in a single case with the clinical diagnosis of persistent Müllerian duct syndrome. We found a genetic alteration in 24 out of 29 (83%) patients (Fig. 2). Among them, 15 individuals (Table 2, cases 33–47) were found to carry a hemizygous mutation in the *AR* gene. Out of 13 identified different sequence changes, six were missense mutations, five were truncating mutations, and two were splicing mutations. The c.1886-2A>G splicing alteration was detected in three unrelated patients (Table 2, cases 42–44). Six identified *AR* alterations were first reported in this study (c.906delC (p.S302Rfs*19), c.1249delG (p.A417Rfs*61), c.1769-13T>G, c.1886-2A>G, c.2290_2291inv.TA (c.(2290A>T;2291T>A), p.Y763I), and c.165_788del (p.L56_L263del)). Eight patients carried a total of 12 different alterations in the *SRD5A2* gene in compound heterozygosity (Table 2, cases 48 and 50–55) or in the homozygous state (Table 2, case 49). Out of 12 mutations, eight were missense, while four were truncating. Three missense mutations recurred in more than one patient: c.513G>C (p.R171S) (Table 2, cases 51 and 52), c.586G>A (p.G196S) (Table 2, cases 50 and 51), and c.1036A>T (p.R246W) (Table 2, cases 50 and 55). The c.513G>C (p.R171S) and the c.763T>C (p.X255Qfs*28) were, respectively, paternally and maternally derived in patient 51. Two of the identified alterations (c.564C>A (p.C188X) and c.332_333delTC (p.L111Hfs*24)) were found to be novel. Finally, a single patient (Table 2, case 56) was a compound heterozygote for a missense (c.367C>T (p.R123W)) and a novel nonsense (c.564C>A (p.C188X)) mutation in the *AMH* gene.

**Figure 3:**
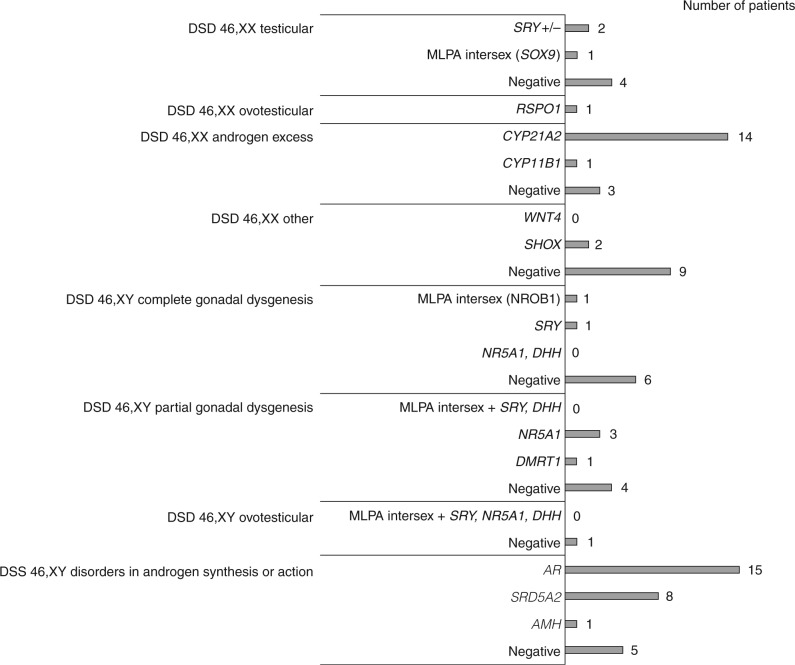
Genetic studies and molecular results for 83 patients with 46,XX and disorders of ovarian development, 46,XX and androgen excess, 46,XX other, 46,XY and disorders of testicular development, and 46,XY and disorders in androgen synthesis or action. DSD clinical classes and genetic tests performed are listed on the *y*-axis. For each molecular analysis, the number of positive (mutated) and negative (not mutated) patients is symbolized by gray bars. *SRY*+/−, SRY presence/absence test; MLPA Intersex: MLPA SALSA P185-B2 Intersex; *RSPO1, CYP21A2, CYP11B1, WNT4, SHOX, SRY, NR5A1, DHH, DMRT1,* and *SRD5A2*, molecular study of the listed genes.

**Figure 4:**
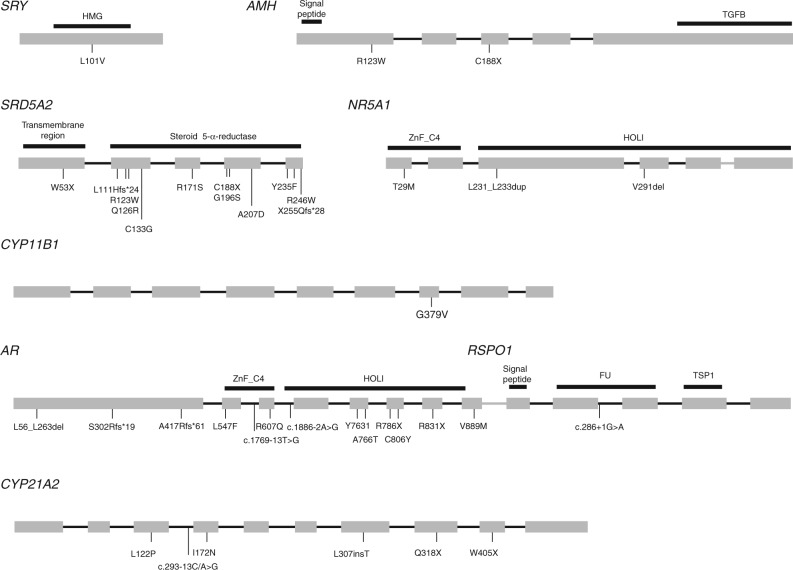
Positions of the identified point mutations along the coding sequences of the *SRY, AMH, SRD5A2, NR5A1, CYP11B1, AR, RSPO1, *and *CYP21A2* genes. Exons are symbolized by gray boxes and introns (not in scale) by black lines. Functional protein domains are represented by black stripes above each gene diagram. HMG, High-Mobility Group; TGFB, transforming growth factor Beta; ZnF_C4, c4 zinc finger; HOLI, ligand-binding domain of hormone receptors; FU, furin-like repeats; TSP1, thrombospondin type 1 repeats. Protein domains were predicted using the SMART software (30).

In total, we found a genetic alteration in 56 out of 88 (64%) patients (Table 2). Figure 3 summarizes the genetic tests, and the respective results, performed on patients with 46,XX disorders of ovarian development, 46,XX androgen excess, 46,XX other, 46,XY disorders of testicular development, and 46,XY disorders in androgen synthesis/action. The positions of all the identified point mutations along the *SRY, AMH, SRD5A2, NR5A1, CYP11B1, AR, RSPO1*, and *CYP21A2* coding sequences are shown in Fig. 4.

## Discussion

DSDs are complex conditions related to a vast number of different causes. Establishing the specific etiology may be crucial for choosing the more adequate sex of assignment, for the clinical management of patients, and to permit the family to plan informed further pregnancies. However, molecular characterization cannot be reached in a consistent number of cases, due to the still limited knowledge of etiological determinants. In this report, we describe the clinical assessment and the cytogenetic and molecular findings in a large cohort of patients with DSD. In our hands, it was possible to identify a genetic defect in 64% of them and to assign each of the examined patients to a specific category in accordance with the current DSD classification. Accordingly, a specific survey could be planned for each patient. This study excluded 45,X as well as 47,XXX and 47,XXY patients and individuals with sex chromosome mosaicism identified during prenatal diagnosis, but that did not display abnormalities of the genital tract after birth. Karyotype analysis performed during this study showed that five out of 88 (6%) patients harbored mosaic sex chromosome anomalies, confirming that standard cytogenetic analyses can detect frequent genetic causes of DSD. Moreover, the initial classification based on clinical and cytogenetic findings was revealed to be an important starting point to carry out the further appropriate molecular testing, specific for each DSD subgroup. Out of 88 patients, 7 (8%) were classified as 46,XX testicular DSD and, between them, two out of seven carried a *SRY* translocation onto the pseudoautosomal region PAR1 of one X chromosome. This aberration is considered the major cause of testicular development in individuals with 46,XX testicular DSD (20, 21, 22). Conversely *SRY* translocation appeared to be involved only in less than a third of our cases. Thus, we assumed that other molecular determinants were responsible for the other 46,XX testicular DSD cases not carrying the *SRY* translocation. The involvement of the *SOX9* gene has already been demonstrated in a number of 46,XX testicular patients with DSD (5, 23, 24). Particularly, Cox *et al*.(24) and Vetro *et al*. (5) reported a 178-kb duplication and a 96 kb triplication, respectively, 600kb and 500 kb upstream of *SOX9*, in 46,XX, *SRY*-negative male patients. These alterations were assumed to enhance the promoter activity leading to *SOX9* overexpression. In this study, we demonstrated the presence of a *SOX9* exon 1 duplication in a 46,XX testicular patient with DSD (Table 2, case 9) not harboring an *SRY* translocation. Although it is not possible to affirm with certainty that the duplication identified in our patient is causative of his DSD, data from the literature permit speculation about its role in the determination of the abnormal gonadal development (5, 23, 24). Owing to the lack of the patient's DNA, it was not possible to investigate whether the rearrangement identified in case 9 extended upstream of *SOX9*, but we cannot exclude the involvement of its promoter. In addition, as patient 9 belongs to north African ethnic group and lives in Africa, DNA neither from other family members nor from healthy controls of his population was available for testing the origin and the possible recurrence of the rearrangement. Our series of *SRY*-negative 46,XX testicular patients with DSD were also investigated for *RSPO1* gene alterations as this gene has already been described as recessively mutated in two familial cases with 46,XX testicular DSD (25). Those patients showed genital anomalies accompanied by additional features, in particular palmoplantar hyperkeratosis. We did not find any *RSPO1* point mutations in our 46,XX testicular DSD cases, implying that the *RSPO1* gene may not be involved in 46,XX testicular DSD without palmoplantar hyperkeratosis. Our series of patients included a single 46,XX ovotesticular DSD case showing palmoplantar hyperkeratosis. This patient was born from consanguineous parents and harbored the c.286+1G>A (p.I32_I95del) *RSPO1 *mutation in homozygosity (12).

The 21-hydroxylase deficiency is considered the most frequent cause of DSD with genital ambiguity. Genetic analysis of *CYP21A2* performed in patients with a definitive or presumptive clinical diagnosis of adrenogenital syndrome allowed the identification of the molecular defect in 14 out of 18 (78%) cases. Among the four negative patients, case 24 was afterward recognized to be affected by a very rare form of congenital adrenal hyperplasia related to 11-β-hydroxylase deficiency. This patient was born as the result of a consanguineous mating and presented ambiguous genitalia at birth. Her clinical and hormonal profile could be defined only after the first weeks of life, when the *CYP21A2* study had already been started. She was found to carry the homozygous p.G379V alteration in the *CYP11B1* gene. The remaining three out of 18 *CYP21A2*-negative patients, in whom adrenogenital syndrome was suspected, showed regression of clitoral hypertrophy throughout the late neonatal period. The evolution of their clinical presentation together with the molecular and hormonal findings led to definitive exclusion of the initial diagnostic hypothesis in these infants.

Regarding 46,XX patients with DSD with abnormal development of the Müllerian structures, our results demonstrated the presence of a duplication involving *SHOX* exon 6 in two out of ten unrelated cases with MRKH syndrome, a condition of still mostly unclear etiology. Nevertheless, our targeted investigations permitted replication of the results obtained from the study by Gervasini *et al*. (4), which reported a *SHOX* intragenic duplication in five patients with abnormal development of the Müllerian ducts. Although the mechanism that may relate *SHOX* duplications and the development of Müllerian ducts has not been clarified, our data and those described by Gervasini *et al*. (4) indicate a possible functional role of *SHOX* in the MRKH syndrome. Based on the results obtained from the study by Philibert *et al*. (26) describing a* WNT4* heterozygous mutation in four cases with MRKH syndrome and hyperandrogenism, we sequenced the *WNT4* gene in 46,XX patients with DSD with abnormal development of the Müllerian structures, but did not find any DNA alteration. In accordance with results from other studies reporting the absence of *WNT4* gene mutations in MRKH women (27, 28), it is possible that the involvement of this gene is restricted to cases with an atypical form of the syndrome.

Investigations of patients affected by 46,XY DSD with a defect in testicular development led to the molecular characterization of six out of 16 (37%) cases. These outcomes are consistent with the still incomplete understanding of the molecular events that underlie testicular development, indicating the need to search for novel genes associated with gonadal dysgenesis in 46,XY patients. The *NR5A1* gene, studied in 46,XY patients with a diagnosis of partial gonadal dysgenesis, was found to be mutated in heterozygosity in 3 out of 8 (37%) cases. These results consistent with those described in recent reports that identify mutations in *NR5A1* as a major cause of 46,XY DSD with a defect in the testicular development. Among the three mutations identified in* NR5A1*, the genetic location of c.86C>T (p.T29M) seems to affect the binding of the protein to DNA, while p.L231_233dup and p.V291 lay in the domain regulating transcription after hormone binding. Interestingly, in the three *NR5A1*-mutated patients, no Müllerian structures seemed to be present. The analysis of the *DHH* gene yielded negative results in all cases of 46,XY DSD with partial gonadal dysgenesis, even if *DHH* alterations have already been described as the possible cause of a consistent number of 46,XY DSD cases with a defect in the testicular development (29).

Concerning patients with 46,XY DSD with a defect in the synthesis or action of androgens, 24 out of 29 (83%) cases were characterized at a molecular level. Among the 12 different identified *SRD5A2 *genetic alterations, one maps in the transmembrane region, possibly affecting the protein localization, and 11 in the protein catalytic domain. Sequence analysis of the *AR *gene identified 13 different mutations, including nine alterations lying in the functional protein domains: three out of nine in the zinc finger domain responsible for the DNA binding, and six out of nine in the domain that regulates the transcription after hormone binding. The incomplete diagnostic sensitivity of the applied molecular studies in 46,XY patients with DSD with a defect in the synthesis or action of androgens might be the cause of the failure of diagnosis in those partial androgen insensitivity syndrome (PAIS) patients for whom negative results were obtained according to *AR* analysis. These patients may indeed harbor DNA alterations in non-canonically investigated *AR* regions (introns or regulatory sequences), or in different known or as yet unidentified genes.

Our results highlight that each category of DSD is related to a large number of different DNA alterations, thus requiring multiple genetic studies to possibly achieve a precise etiological diagnosis in every patient. Currently, as a consequence of the incomplete knowledge concerning the genetic factors involved in the differentiation of testes and ovaries, DSD associated with anomalies in gonadal development still often lacks a molecular diagnosis. A multidisciplinary and specialized DSD center is the key for the correct clinical management of neonates in cases of ambiguous genitalia. Moreover, the introduction of new technologies for massive parallel sequencing is becoming helpful for the molecular characterization of patients with DSD by analyzing previously known genes as well as candidate genes.

## Supplementary data

This is linked to the online version of the paper at http://dx.doi.org/10.1530/EC-14-0085.

## Author contribution statement

L Laino participated in the design and coordination of the study, carried out the cytogenetic studies, and drafted the manuscript; S Majore provided the genetic counseling to the patients, established the clinical diagnosis, participated in the study design, and helped to draft the manuscript; N Preziosi carried out the molecular genetic studies; B Grammatico participated in the design and helped to draft the manuscript; C De Bernardo participated in the design and helped to draft the manuscript; S Scommegna established the clinical diagnosis, participated in the study design, and helped to draft the manuscript; A M Rapone provided the psychological support to the families of the patients; G Marrocco conceived the study and gave the final approval of the version to be published; I Bottillo conceived the study, drafted the manuscript, and gave the final approval of the version to be published; and P Grammatico conceived the study and gave the final approval of the version to be published. All authors read and approved the final manuscript.

## Figures and Tables

**Table 1:** Classification of the studied patients with DSD (based on Hughes *et al.* (1))

	
Sex chromosome DSDs	45,X (Turner's syndrome and variants)
	47,XXY (Klinefelter syndrome and variants)
	45,X/46,XY (mixed gonadal dysgenesis and ovotesticular DSD)
	46,XX/46,XY (chimeric and ovotesticular DSD)
DSD 46,XX disorders of gonadal (ovarian) development	Ovotesticular DSDs
	Testicular DSDs
	Gonadal dysgenesis
DSD 46,XX androgen excess	Fetal 21 hydroxylase deficiency
	Fetal 11 hydroxylase deficiency
	Fetoplacental aromatase deficiency
	Maternal luteoma
	Exogenous androgen excess
DSD 46,XX other	MURCS, MRKH, and other syndromes
DSD 46,XY disorders of gonadal (testicular) development	Complete gonadal dysgenesis (Swyer syndrome)
	Partial gonadal dysgenesis
	Gonadal regression
	Ovotesticular DSDs
DSD 46,XY disorders in androgen synthesis or action	Androgen biosynthesis defect (5-α reductase deficiency)
	Defect in androgen action (androgen insensitivity syndrome)
	Disorders of *AMH* and *AMH* receptor (persistent Müllerian duct syndrome)

MURCS, Mullerian Renal Cervicothoracic Somite association; MRKH, Mayer Rokitansky Küster Hauser syndrome.

**Table 2:** Clinical and genetic features of DSD mutated patients. The mutations not marked by any symbol were identified in heterozygosity/hemizygosity

**Karyotype**	**Disorder of sex development**		**Patient**	**Genes**	**Mutations**	**References**
mos 45,X(7)/47,X,idic(Y)(q12),+mar.ish der(Y)(DYZ1+)(17)/46,X,idic(Y)(q12)(76)	Sex chromosome		1			
mos 46,X(20)/45,XY(80)			2			
mos 45,X(70)/46,X,idic(Y)(qter→p11.3::p11.3→qter)(30)			3			
45,X,(51)/46,X,idic(Y)(q11.23)(49)			4			
45,X(23)/46,XY(77)			5			
46,XX	Disorders of ovarian development	Ovotesticular	6	*RSPO1*	c.286+1G>A (p.I32_l95del)^§^	(12)
46,XX.ish der(X)(X;Y)(p22.3;p11.3)(SRY+)		Testicular	7, 8	*SRY*	Entire gene traslocation to chrX	(20)
46,XX			9	*SOX9*	Exon 1 duplication	This report
46,XX	Androgen excess	Fetal (21 hydroxylase deficiency)	10	*CYP21A2*	c.293-13C/A>G^§^	(31)
11	*CYP21A2*	del/conv	(16)
					c.515T>A (p.I172N)	(13)
			12, 13, 14	*CYP21A2*	del/conv^§^	(16)
			15, 16, 17	*CYP21A2*	del/conv	(16)
					c.293-13C/A>G	(17)
			18	*CYP21A2*	c.293-13C/A>G	(17)
					c.1214G>A (p.W405X)	(15)
			19	*CYP21A2*	del/conv	(16)
					c.365T>C (p.L122P)	This report
			20	*CYP21A2*	c.515T>A (p.I172N)^§^	(13)
			21	*CYP21A2*	c.952C>T (p.Q318X)^§^	(18)
			22	*CYP21A2*	c.293-13C/A>G	(17)
					c.920_921insT (p.L307insT)	(31)
			23	*CYP21A2*	del/conv	(16)
					c.952C>T (p.Q318X)^§^	(18)
		Fetal (11 hydroxylase deficiency)	24	*CYP11B1*	p.G379V^§^	(14)
46,XX	Other	MRKH	25	*SHOX*	Exon 6 duplication	This report
			26	*SHOX*	Exon 6 duplication	This report
46,XY	Disorders of testicular development	Complete gonadal dysgenesis	27	*NR0B1*	Entire gene duplication	(32)
			28	*SRY*	c.301C>G (p.L101V)	(33)
		Partial gonadal dysgenesis	29	*NR5A1*	c.691_699 dupCTACAGCTG (p.L231_L233dup)	(19)
			30	*NR5A1*	c.86C>T (p.T29M)	This report
			31	*NR5A1*	c.872_874delTGG (p.V291del)	This report
			32	*DMRT1*	Entire gene deletion	(34)
46,XY	Disorders in androgen synthesis or action		33	*AR*	c.1641C>G (p.L547F)	(35)
	34	*AR*	c.2290_2291inv.TA (c.(2290A>T;2291T>A), p.Y763I)	This report
			35	*AR*	c.2491C>T (p.R831X)	(36)
			36	*AR*	c.2718C>T (p.R786X)	(37)
			37	*AR*	c.2417G>A (p.C806Y)	(38)
			38	*AR*	c.2665G>A (p.V889M)	(39)
			39	*AR*	c.1249delG (p.A417Rfs*61)	This report
			40	*AR*	c.1820G>A (p.R607Q)	(40)
			41	*AR*	c.2296G>A (p.A766T)	(41)
			42, 43, 44	*AR*	c.1886-2A>G	This report
			45	*AR*	c.906delC (p.S302Rfs*19)	This report
			46	*AR*	c.1769-13T>G	This report
			47	*AR*	c.165_788del (p.L56_L263del)	This report
			48	*SRD5A2*	c.158G>A (p.W53X)	(42)
					c.704A>T (p.Y235F)	(43)
			49	*SRD5A2*	c.397T>G (p.C133G)^§^	(44)
			50	*SRD5A2*	c.586G>A (p.G196S)	(45)
					c.1036A>T (p.R246W)	(46)
			51	*SRD5A2*	c.513G>C (p.R171S)	(47)
					c.763T>C (p.X255Qfs*28)	(48)
			52	*SRD5A2*	c.513G>C (p.R171S)	(47)
					c.586G>A (p.G196S)	(46)
			53, 54	*SRD5A2*	c.332_333delTC (p.L111Hfs*24)	This report
					c.620C>A (p.A207D)	(49)
			55	*SRD5A2*	c.377A>G (p.Q126R)	(47)
					c.1036A>T (p.R246W)	(46)
			56	*AMH*	c.367C>T (p.R123W)	(50)
					c.564C>A (p.C188X)	This report

MRKH, Mayer-Rokitansky-Kuster-Hauser; del/conv, deletion/conversion.

§ Homozygous mutations. The mutations not marked by any symbol were identified in heterozygosity/hemizygosity.
